# P-344. Modeling Confirms Islatravir 0.25 mg Administered Daily Has No Adverse Effect on Total Lymphocyte or CD4+ T-cell Counts in People Living with HIV

**DOI:** 10.1093/ofid/ofaf695.562

**Published:** 2026-01-11

**Authors:** Brian M Maas, Bill Poland, Michelle M Pham, Martin Johnson, Irene Bae, Seth H Robey, Bhargava Kandala, Xiaowei Zang, Stephanie O Klopfer, Michelle C Fox, Ryan C Vargo

**Affiliations:** Merck & Co., Inc., Rahway, NJ; Certara, Princeton, New Jersey; Merck & Co., Inc., Rahway, NJ; Allucent, Bracknell, England, United Kingdom; Certara, Princeton, New Jersey; Merck & Co., Inc., Rahway, NJ; Merck & Co., Inc., Rahway, NJ; Merck & Co., Inc., Rahway, NJ; Merck & Co., Inc., Rahway, NJ; Merck & Co., Inc., Rahway, NJ; Merck & Co., Inc., Rahway, NJ

## Abstract

**Background:**

Islatravir (ISL) is an investigational nucleoside reverse transcriptase translocation inhibitor being evaluated in combination regimens for the treatment of HIV-1. Exposure-dependent decreases in total lymphocytes count (TLC) and CD4+ T-cells (CD4s) were previously observed in clinical development. Pharmacokinetic-pharmacodynamic (PK-PD) models predicted no changes in TLC or CD4s with ISL 0.25 mg once daily (QD), leading to reinitiation of the Phase 3 program with doravirine (DOR). The objectives of this work were to 1) update the previous models with data from the oral DOR/ISL (100/0.25 mg) QD Phase 3 program in virologically suppressed participants, 2) predict TLC and CD4 changes at 48 weeks with ISL 0.25 mg, and 3) identify the ISL-triphosphate (ISL-TP) exposure associated with CD4 changes similar to those with approved antiretroviral therapy (ART).

Figure

**Figure:**
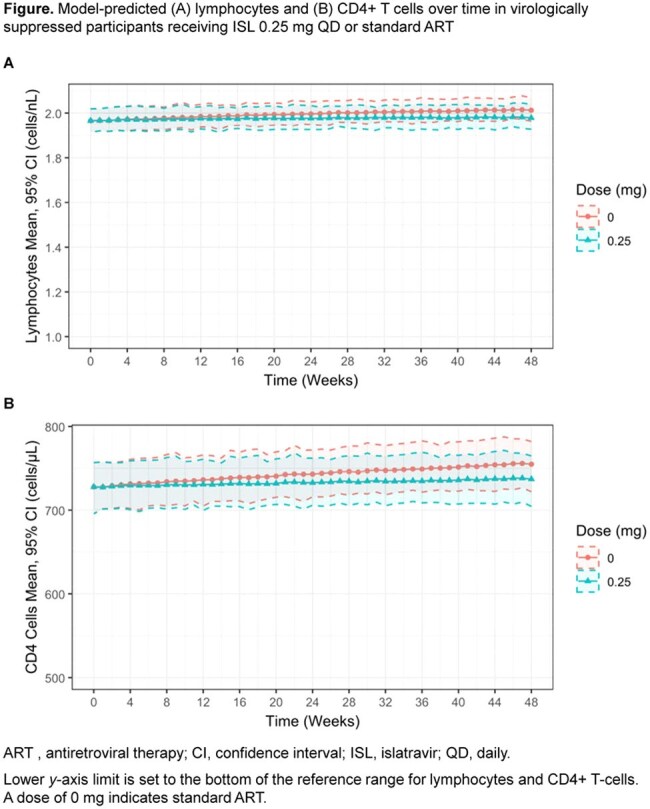

**Methods:**

PK-PD dynamics were characterized using indirect response models, where ISL-TP concentrations increased the rate of TLC and CD4 loss. PK-PD models for TLC and CD4 were updated with data from the DOR/ISL (100/0.25 mg) QD Phase 3 program. The models were used to predict changes in TLC and CD4 at 48 weeks and were compared with the observed Phase 3 data. ISL-TP exposure associated with changes in CD4s comparable to that of approved ART (ie, ≤ 5% decrease) was predicted.

**Results:**

PK-PD models for TLC and CD4 adequately described observed data in virologically suppressed participants across the range of doses evaluated in the clinical program. No intrinsic factors impacted the relationship between ISL-TP exposure and changes in TLC or CD4s. ISL 0.25 mg QD was predicted to result in TLC and CD4 changes at Week 48 that were similar to those with standard ART in virologically suppressed participants (Figure). Model predictions suggest changes in CD4 were comparable to approved ART up to ISL exposures that were 1.8-fold higher than those produced by ISL 0.25 mg QD.

**Conclusion:**

Consistent with Phase 3 clinical studies, updated modeling indicates that ISL 0.25 mg administered QD results in TLC and CD4 changes that are similar to those with standard ART. No reductions in CD4s are expected with exposures up to almost 2-fold higher than the proposed clinical dose of ISL 0.25 mg.

**Disclosures:**

Brian M. Maas, PharmD, Merck & Co. Inc.: Employment|Merck & Co. Inc.: Stocks/Bonds (Public Company) Michelle M. Pham, PhD, Merck & Co., Inc: Full Time employee Martin Johnson, PhD, Allucent: Stocks/Bonds (Public Company) Seth H. Robey, PhD, Merck & Co.,Inc.: Employment|Merck & Co.,Inc.: Stocks/Bonds (Public Company) Bhargava Kandala, PhD, Merck & Co.: Employment|Merck & Co.: Stocks/Bonds (Public Company) Xiaowei Zang, PhD, Merck & Co., Inc.: Employment|Merck & Co., Inc.: Stocks/Bonds (Public Company) Stephanie O. Klopfer, PhD, Merck & Co., Inc: Employment|Merck & Co., Inc: Stocks/Bonds (Public Company) Michelle C. Fox, MD, Merck & Co., Inc.: Employment|Merck & Co., Inc.: Stocks/Bonds (Public Company) Ryan C. Vargo, PhD, Merck and Co.: Full time employee|Merck and Co.: Stocks/Bonds (Public Company)

